# Carrot-Derived Rhamnogalacturonan-I Consistently Increases the Microbial Production of Health-Promoting Indole-3-Propionic Acid Ex Vivo

**DOI:** 10.3390/metabo14120722

**Published:** 2024-12-21

**Authors:** Annick Mercenier, Lam Dai Vu, Jonas Poppe, Ruud Albers, Sue McKay, Pieter Van den Abbeele

**Affiliations:** 1 NutriLeads BV, 6708 WH Wageningen, The Netherlands; annick.mercenier@nutrileads.com (A.M.); ruud.albers@nutrileads.com (R.A.); sue.mckay@nutrileads.com (S.M.); 2 Cryptobiotix SA, 9052 Ghent, Belgium; lamdai.vu@cryptobiotix.com (L.D.V.); jonas.poppe@cryptobiotix.com (J.P.)

**Keywords:** indole-3-propionic acid, tryptophan metabolism, *Bifidobacterium longum*, carrot rhamnogalacturonan-I (cRG-I), dietary fiber, prebiotic, ex vivo

## Abstract

Background: Using dietary interventions to steer the metabolic output of the gut microbiota towards specific health-promoting metabolites is often challenging due to interpersonal variation in treatment responses. Methods: In this study, we combined the ex vivo SIFR^®^ (Systemic Intestinal Fermentation Research) technology with untargeted metabolite profiling to investigate the impact of carrot-derived rhamnogalacturonan-I (cRG-I) on ex vivo metabolite production by the gut microbiota of 24 human adults. Results: The findings reveal that at a dose equivalent to 1.5 g/d, cRG-I consistently promoted indole-3-propionic acid (IPA) production (+45.8% increase) across all subjects. At a dose equivalent to 0.3 g/d, increased IPA production was also observed (+14.6%), which was comparable to the effect seen for 1.5 g/d inulin (10.6%). IPA has been shown to provide protection against diseases affecting the gut and multiple organs. The Pearson correlation analysis revealed a strong correlation (R = 0.65, *p*_adjusted_ = 6.1 × 10^−16^) between the increases in IPA levels and the absolute levels of *Bifidobacterium longum*, a producer of indole-3-lactic acid (ILA), an intermediate in IPA production. Finally, the community modulation score, a novel diversity index, demonstrated that cRG-I maintained a high α-diversity which has previously been linked to elevated IPA production. Conclusions: The results from the ex vivo SIFR^®^ experiment mirrored clinical outcomes and provided novel insights into the impact of cRG-I on the gut microbiome function. Importantly, we demonstrated that cRG-I promotes tryptophan conversion into IPA via gut microbiome modulation, thus conferring benefits via amino acid derived metabolites extending beyond those previously reported for short chain fatty acids (SCFA) resulting from carbohydrate fermentation.

## 1. Introduction

The gut microbiome is a complex community of microorganisms residing in the gastrointestinal tract that plays a crucial role in maintaining host health by producing a variety of metabolites that contribute to maintaining intestinal homeostasis, gut barrier integrity as well as modulating immune responsiveness under inflammatory conditions [1,2]. In addition, a plethora of gut microbial metabolites is absorbed into the bloodstream, reaching other organs and forming regulatory “gut-organ axes” that mediate interactions between the gut microbiota and these organs [3]. Among the metabolites produced by the gut microbiota, tryptophan-derived metabolites have gained a lot of attention for their potential health benefits [4]. For example, indole-3-propionic acid (IPA) and its precursor indole-3-lactic acid (ILA) can activate aryl hydrocarbon receptor (AhR)-mediated signaling and induce the production of anti-inflammatory interleukins [5,6,7]. The strong antioxidant and anti-inflammatory activity of IPA is not limited to the gut but can extend to multiple gut–organ axes, so that IPA has been attributed therapeutic potential for, e.g., the cardiovascular system, metabolic health, and neuroprotection [8,9].

Since diet is an important factor in shaping the gut microbiota, dietary interventions, especially with functional carbohydrates such as prebiotics can be used to influence its composition and metabolic activity [10]. By definition, prebiotics are indigestible carbohydrates that are selectively utilized by beneficial microbes present in the gut [11]. The health effects of dietary fibers vary vastly depending on their biochemical composition [12] and on the complexity of their structure that underlies the specificity of their effect on the gut microbiome [13,14]. Recently, carrot-derived rhamnogalacturonan-I (cRG-I), a medium specificity fiber, was shown to lower interpersonal compositional differences across a cohort of 24 healthy adults ex vivo due to the selective stimulation of bacteria that were consistently present in all test subjects [15]. In contrast, the reference prebiotic inulin (IN) and xanthan (XA) increased interpersonal differences due to the low specificity and very high specificity of their effects on the microbiome, respectively. Additionally, cRG-I induced a higher production of short-chain fatty acids (SCFAs) compared to IN and XA, potentially delivering local benefits for gut health by strengthening the intestinal epithelial barrier [16]. SCFAs can also benefit more remote locations after entering the systemic circulation [17]. However, the impact of cRG-I on the production of other health-related microbial metabolites beyond SCFAs has not yet been investigated.

In addition to metabolites derived directly from fiber fermentation such as SCFAs, it is widely known that carbohydrates can also indirectly influence the utilization of other substrates, such as amino acids. In particular, the supplementation of specific dietary fibers and polyphenols can increase the production of various beneficial tryptophan metabolites, including ILA and IPA [18,19]. However, reshaping the microbial composition or modulating different metabolic pathways, through dietary interventions, to stimulate consistent specific health-related metabolites still remains a challenge.

In this study, untargeted metabolite profiling was performed on samples derived from an earlier ex vivo SIFR^®^ colonic fermentation of cRG-I, IN and XA [15] to add further insights into metabolites produced beyond SCFA. Importantly, applying untargeted metabolomics revealed that cRG-I consistently increased the production of the health-promoting IPA by the gut microbiota from 24 human adults. The increased IPA levels strongly correlated with the prevalence of an OTU related to *Bifidobacterium longum* which converts tryptophan to ILA, a precursor of IPA. These findings suggest that cRG-I may promote the reductive Stickland conversion of tryptophan [19] by consistently increasing the abundance of a bacterial taxon involved in this pathway.

## 2. Materials and Methods

### 2.1. Test Compounds

The test compounds evaluated in this study were IN from chicory (I2255, Merck, Overijse, Belgium), XA (3557, Carl Roth, Karlsruhe, Germany), and cRG-I (Benicaros^®^, NutriLeads, Wageningen, The Netherlands). Indigestibility of cRG-I in the human upper gastro-intestinal tract has been previously demonstrated [20]. More details of the test compounds have been described by Van den Abbeele et al., 2023 [15].

### 2.2. SIFR^®^ Colonic Incubation and Microbial Composition Analysis via Quantitative 16S rRNA Gene Profiling

**Figure 1:**
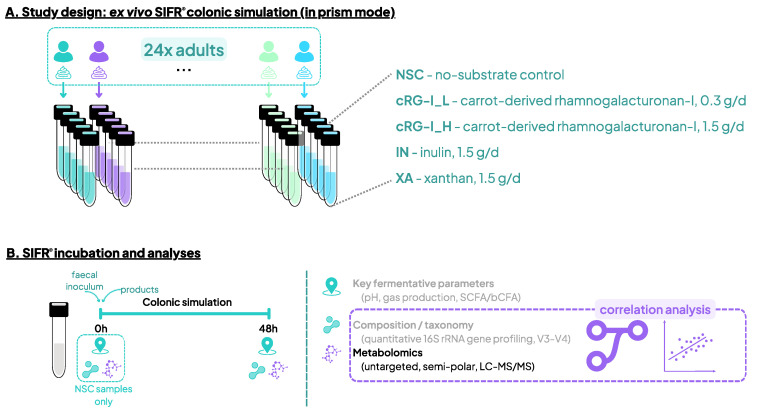
Study design using the ex vivo SIFR^®^ technology to assess the impact of cRG-I, IN and XA on the human gut microbiota. (**A**) Reactor design using the ex vivo SIFR^®^ technology to test the impact of the fibers with different specificities at a dose equivalent to 0.3 g/d (cRG-I_L) or 1.5 g/d (cRG-I_H, IN and XA), compared to a no-substrate control (NSC) in fecal samples of 24 human adults in parallel. (**B**) Timeline and analyses at 0 h and 48 h. Analysis of key fermentation parameters and microbial composition was reported earlier by Van den Abbeele et al., 2023 [15].

The ex vivo SIFR^®^ technology was developed to study the human gut microbiota across numerous parallel test conditions (both treatments and test subjects). It has been validated to provide predictive insights into the impact of various substrates on microbial composition [21], gut barrier integrity and immunity [22]. Experimental procedures are described in detail by Van den Abbeele et al., 2023 [15]. Briefly, five experimental conditions were tested for 24 human adults: a no-substrate control (NSC), a low dose equivalent to 0.3 g/d of cRG-I (cRG-I_L), 1.5 g/d cRG-I (cRG-I_H), 1.5 g/d inulin (IN), and 1.5 g/d of xanthan (XA) (Figure 1A). Fecal fermentation of test products as simulated by SIFR^®^ technology and analysis of microbial composition Via 16S rRNA gene profiling was performed in the previous study (Figure 1B) [15]. The 24 test subjects that provided a fecal sample (13 males, 11 females) were selected based on the following criteria: age 25–65 years (average age of 38.5 years), no antibiotics use in the past 3 months, no gastrointestinal disorders, no probiotic use, nonsmoking, alcohol consumption < 3 units/day, and BMI < 30 [15].

### 2.3. Untargeted Metabolite Profiling

After removing insoluble particles and surfactants present in the fecal fermentation samples, the samples were diluted 11-fold in buffer consisting of 10 mM ammonium formate and 0.1% formic acid. Before analysis, stable isotope-labeled standards were added to the diluted samples. Mass spectrometry analysis has been previously described in detail [23]. Technical variability was confirmed by running a quality control sample (pooled sample of all samples) every six samples. Coefficients of variation for these quality control samples were, on average, 8.2% for level 1/2a-annotated metabolites, confirming the high technical reproducibility of the method.

### 2.4. Calculation of Community Modulation Score

The community modulation score (CMS) was calculated as described by Tintoré et al., 2024 [24], based on the compositional analysis of the preceding study [15]. In short, the CMS represents the number of OTUs (out of the 100 most abundant OTUs) that increased (positive CMS) or decreased (negative CMS) upon treatment. A combined CMS has a positive value when the number of species that are increased exceeds the number of species that are decreased, suggesting that a treatment increases the gut microbiota diversity.

### 2.5. Statistical Analysis

Univariate and multivariate analyses were performed using R (version 4.4.0; www.r-project.org; accessed on 24 April 2024). Given the higher degree of certainty of correct annotation at level 1 and 2a, statistical analysis was only performed on level 1/2a metabolites. An additional filter was applied to retain metabolites that increased within 48 h compared to the initial concentration in the no-substrate control at 0 h. These metabolites were thus either produced by human-derived gut microbes or alternatively already present in one of the test products. Benjamani–Hochberg correction was applied within each comparison (NSC vs. 4 treatments), given the large number of features analyzed.

Regularized canonical correlation analysis (rCCA) was performed to highlight correlations between metabolites and compositional data (at family and OTU level), using the mixOmics package with the shrinkage method for estimation of penalization parameters (version 6.28.0) in R (4.1.1; www.r-project.org; accessed on 24 June 2024) [25]. The Benjamani–Hochberg correction was followed to calculate the false discovery rate. In addition, pairwise correlation analysis based on the Pearson correlation coefficient was also performed for the significantly affected metabolites and the significantly affected OTUs.

## 3. Results

### 3.1. cRG-I, IN and XA Affected Microbial Metabolite Production According to Previously Established Fiber Specificity

**Figure 2:**
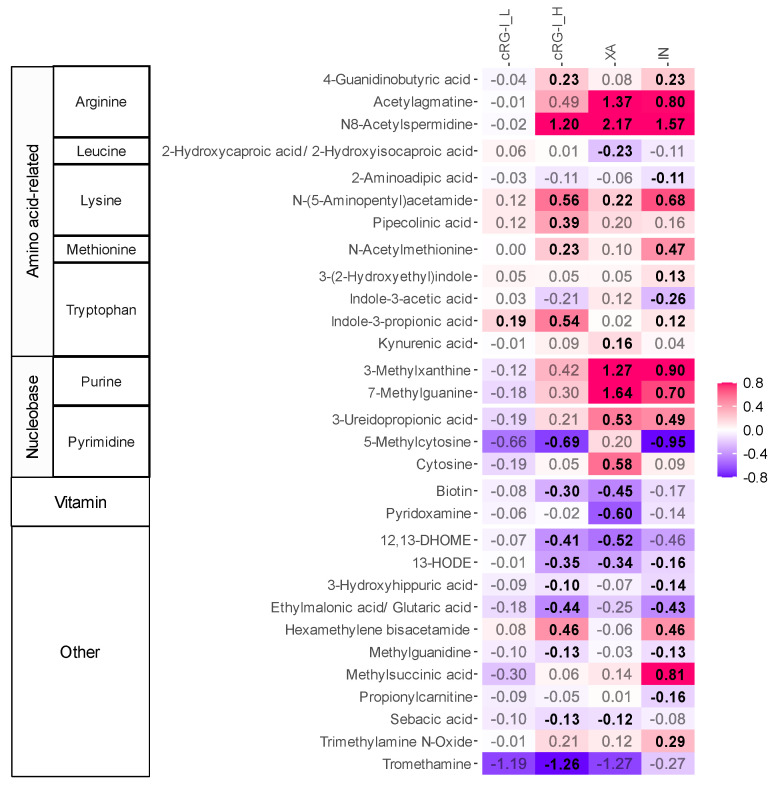
cRG-I, XA and IN stimulated the microbial production of different metabolites. The heat map displays the impact of a dose equivalent to 0.3 g/d carrot-derived rhamnogalacturonan-I (cRG-I_L) or 1.5 g/d carrot-derived rhamnogalacturonan-I (cRG-I_H), inulin (IN) and xanthan (XA) on a selection of metabolites identified at level 1 and 2a, as quantified Via untargeted LC-MS after 48 h of incubation. Colonic fermentation was simulated using SIFR^®^ technology for healthy adults (*n* = 24). The reported metabolites were significantly affected by the treatments (FDR < 0.20). Significant differences are indicated in bold of the log_2_-transformed average fold change (abundance treatment/abundance NSC). Metabolite classes and subclasses (based on the precursor amino acids or nucleobases) are indicated on the left side of the heat map. cRG-I: carrot-derived rhamnogalacturonan-I, IN: inulin, XA: xanthan.

Untargeted metabolite profiling revealed substrate-specific effects on metabolite production (Figure 2). Overall, after 48 h of fermentation, the effects of the three fibers on metabolite production reflected their previously reported specificity (low, medium, or high) [15], i.e., their selective impact on microbial composition.

First, at a dosage of 1.5 g/d, the fermentation of XA, a high-specificity fiber, resulted in a strong increase in the levels of acetylagmatine and N8-acetylspermidine, which are derivatives from the decarboxylation of arginine. Another class of metabolites strongly promoted by XA were those derived from heterocyclic aromatic amines such as purine and pyrimidine, with significant increases in methylated purines like 3-methylxanthine and 7-methylguanine. Conversely, the fermentation of XA significantly reduced the production of biotin (vitamin B7) and pyridoxamine (vitamin B6), as well as the linoleic acid derivatives 12,13-dihydroxy-9Z-octadecenoic acid (12,13-DiHOME) and 13-Hydroxyoctadecadienoic acid (13-HODE). Like its highly specific effects on microbial composition, the impact of XA on metabolite production was also very selective, markedly enhancing and suppressing only a few classes of metabolites.

Compared to XA, the fermentation of cRG-I_H (1.5 g/d), previously identified as a medium specificity fiber [15], stimulated the production of a wider range of metabolites, which was largely consistent across test subjects. In addition to increasing levels of N8-acetylspermidine, cRG-I_H also stimulated the production of 4-guanidobutyric acid, a derivative of arginine and the neurotransmitter γ-aminobutyric acid (GABA). Furthermore, cRG-I_H significantly boosted the production of metabolites from lysine (N-(5-aminopentyl)acetamide and pipecolinic acid), tryptophan (IPA), and methionine (N-acetylmethionine). Additionally, cRG-I_H significantly enhanced levels of hexamethylene bisacetamide (HMBA). While not significant, cRG-I_H tended to elevate levels of the two methylated purines that were also stimulated by XA. At the lower dose (0.3 g/d), cRG-I_L displayed a similar profile to cRG-I_H. However, the effects were only significant for IPA, potentially due to the small effect size of a low dose.

The fermentation of IN, a low-specificity fiber, resulted in the production of the broadest range of metabolites. However, the size of the effects was often smaller compared to XA and/or cRG-I. Unlike XA and cRG-I, IN specifically increased methylsuccinic acid and trimethylamine N-oxide (TMAO).

### 3.2. Correlation Analysis Confirmed Estalished Links Between Microbial Composition and Metabolite Production

Next, correlations between changes in microbial composition and metabolite production were analyzed by rCCA (Figure S1), which nicely confirmed several previously observed correlations, e.g., those between *Collinsella aerofaciens* (OTU6) and indole-3-acetic acid (IAA) [26] or between *Bifidobacterium adolescentis* (OTU3) and acetylagmatine [27], kynurenic acid [28] as well as biotin [29]. Strikingly, a novel correlation observed at both taxonomic levels was the one between *Bifidobacteriaceae*, specifically *Bifidobacterium longum* (OTU10), and enhanced levels of IPA (see Section 3.3 for detailed analysis).

### 3.3. Fermentation of cRG-I Enhances Production of Beneficial Metabolites and Reduces the Levels of Disease-Associated Linoleic Acid Derivatives

**Figure 3:**
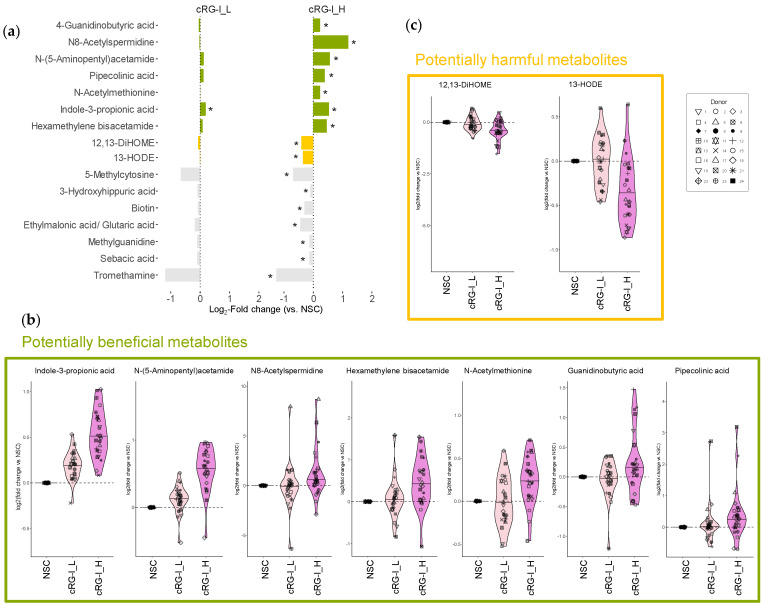
cRG-I enhanced the microbial production of health-related metabolites and reduced the production of harmful linoleic acid derivatives. (**a**) The bar chart showing level 1/2a metabolites that were significantly affected (highlighted by asterisks) by an equivalent dose of 0.3 and 1.5 g/d carrot-derived rhamnogalacturonan-I (cRG-I_L and cRG_H, respectively), after 48 h of SIFR^®^ colonic incubation for healthy adults (*n* = 24). The data are presented as log_2_-transformed average fold change (abundance treatment/abundance NSC). Potentially beneficial and harmful metabolites are highlighted in green and yellow, respectively, while metabolites in gray are not discussed with respect to health benefits. (**b**) log2-transformed fold change versus NSC for a selection of health-related metabolites promoted by cRG-I. (**c**) Disease-associated linoleic acid derivatives that were reduced by cRG-I.

The effects of cRG-I revealed potential benefits for the host through the production of specific metabolites (Figure 3a,b). Notably, IPA levels already significantly increased at the lower cRG-I dose of 0.3 g/d, indicating enhanced Stickland conversion of tryptophan. Higher levels of IPA, a metabolite with strong anti-inflammatory and antioxidant properties, may enhance gut barrier function and protect various organs, including the brain, heart, liver, lungs, kidneys, and skeletal muscles from disease [8,9]. Additionally, the elevated levels of N8-spermidine and N-(5-aminopentyl)acetamide (or N-acetylcadaverine) suggest augmented activity in polyamine biosynthesis pathways, which are associated with cardioprotective, neuroprotective, and anticancer effects and may also promote longevity [30,31]. Moreover, cRG-I_H increased levels of HMBA, a di-acetylated form of hexamethylenediamine, known for its therapeutic potential in treating cancer [32], bacterial infections [33] as well as anti-obesity effects such as weight loss [34]. Other notable metabolites that were more abundant with health benefits preclinically demonstrated in animal models include N-acetylmethionine reported to display antioxidant properties [35], 4-guanidinobutyric acid that showed inhibitory effects against gastric lesions [36], and pipecolinic acid (also called pipecolic acid) which has been shown to alleviate constipation [37].

In addition to promoting beneficial metabolites, the fermentation of cRG-I_H significantly decreased the levels of several metabolites, particularly 12,13-DiHOME and 13-HODE which are derived from the oxidation of linoleic acid and are potentially detrimental to health (Figure 3c). 12,13-DiHOME, also known as isoleukotoxin diol, exhibits cytotoxicity, which contributes to its immunosuppressive properties [38]. For example, elevated levels of 12,13-DiHOME produced by the gut microbiota can impede the development of immune tolerance in neonates and may play a role in increasing risk of asthma in humans [39]. Similarly, 13-HODE is also a clinically relevant lipid potentially associated with various diseases, among others, liver steatosis [40], asthma [41] and various cancer types [42,43,44]. Therefore, the inhibition of these harmful metabolites may contribute to the potential health benefits of cRG-I supplementation.

### 3.4. cRG-I Consistently Stimulated IPA Production and Increased Abundance of Bifidobacterium longum

**Figure 4:**
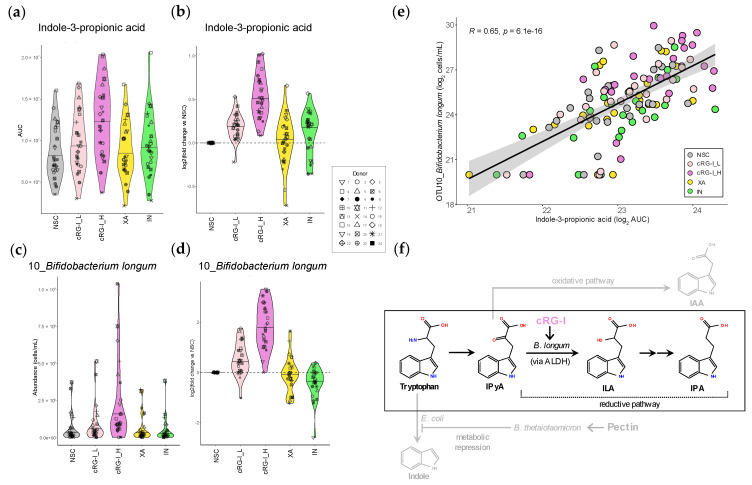
The fermentation of cRG-I promoted indole-3-propionic acid (IPA) production consistently across 24 donors, correlating with the consistent increase in *Bifidobacterium longum* (OTU10). (**a**) Absolute IPA levels (area under curve, AUC) and (**b**) log_2_-transformed fold change versus NSC, as quantified Via LC-MS after 48 h SIFR^®^ colonic fermentation of carrot-derived rhamnogalacturonan-I (cRG-I), inulin (IN) and xanthan (XA) by the gut microbiota of 24 healthy adults. (**c**) Absolute levels (cells/mL) and (**d**) log_2_-transformed fold change in *B. longum* (OTU10). (**e**) The Pearson correlation analysis between *B. longum* (OTU3) and IPA across all study arms. The Pearson correlation coefficient (R) and corrected *p*-value indicating the significance of the correlation are presented. (**f**) Schematic presentation of reductive conversion of tryptophan into IPA Via indole-3-pyruvic acid (IPyA) and indole-3-lactic acid (ILA). cRG-I likely promotes IPA Via stimulation of ILA-producing *B. longum*. Competing pathways that convert tryptophan to indole-3-acetic acid (IAA) and indole are shown in gray. Interactions between *B. thetaiotaomicron* and *E. coli* that suppress indole biosynthesis upon pectin supplementation are also shown [19].

The effects on the production of the health-promoting tryptophan derivative IPA were examined in more detail. First, inspection of the MS/MS spectrum confirmed correct annotation of IPA (Figure S2). Then, the remarkable finding that IPA was the only metabolite that was significantly increased by the low dose of cRG-I_L (+14.6%), an effect comparable to the increase observed with IN (+10.6%) (Figure 4a). This became even more apparent when plotting the log_2_-transformed fold change versus NSC for individual donors (Figure 4b). This illustrated that levels of IPA consistently increased with cRG-I_H for all 24 adults (+45.8% increase) (Figure 4b). In comparison, at the same dose, the fermentation of IN resulted in a lower increase in IPA levels, and an increase was not observed for all donors.

The strong positive correlation between IPA and *B. longum* (OTU10) identified through the rCCA analysis (Figure S1) was examined in more detail. cRG-I strongly enhanced the absolute abundance of *B. longum* (OTU10), already at the low dose of 0.3 g/d (Figure 4c,d), which notably was equal or superior to 1.5 g/d of IN or XA, respectively. This effect also became consistent across all but one donor (donor 14) at the higher dose of cRG-I. Interestingly, *B. longum* was absent in the microbiota of donor 14 at baseline. This also correlated with the lowest IPA levels across all study arms and the smallest increase in IPA production with cRG-I_H for this donor. The Pearson correlation coefficient analysis further confirmed a significant correlation between *B. longum* (OTU10) and IPA across all study arms (R = 0.65) (Figure 4e). cRG-I also enhanced the absolute levels of an OTU related to another *Bifidobacterium* species, *B. adolescentis* (OTU3). Notably, the positive correlation between *B. adolescentis* (OTU3) and IPA was much weaker (R = 0.32) than the correlation between IPA and *B. longum* (Figure S3). Altogether, these findings suggest that *B. longum* is a key taxon contributing to IPA production by the human gut microbiota upon cRG-I supplementation.

## 4. Discussion

Nutritional interventions with dietary fibers (including prebiotics) have often presented inconsistent results. The poor reproducibility in these results in human trials is commonly considered to be due to the inter-individual variability of the gut microbiome composition. Cantu-Jungles et al. recently advocated that the structural complexity of the dietary fiber may also impact the outcome and thus the consistency of the results of nutritional trials [13,14]. Low specificity fibers with a simple structure, i.e., single sugar composition and repetitive linkages, will serve as a substrate for a large variety of bacteria pre-existing in the gut microbiota, thus leading to variable, host-dependent, effects. Whereas the fermentation of medium to high specificity fibers—with more complex structures, i.e., with multiple monosaccharides and linkages—require and stimulate the concerted action of specific microbial communities.

In a previous ex vivo study we demonstrated that cRG-I, a fiber with medium specificity, remarkably reduced interpersonal differences in microbial composition upon fermentation by the gut microbiota of 24 adults representing different enterotypes [15]. In contrast, these interpersonal differences were increased by dietary fibers with low (IN) or very high specificity (XA) [15]. Notably, the capacity of cRG-I to reduce inter-individual gut microbial variation was confirmed in a human clinical trial [45]. In the current study, untargeted metabolite profiling revealed distinct impacts of IN and XA on the colonic microbial metabolome within the same cohort. Although both XA and IN significantly enhanced the production of several health-promoting metabolites, none of these metabolites were consistently stimulated across all 24 test subjects. This variability again underscores the potentially less predictable health outcomes associated with the supplementation of dietary fibers with low or overly high specificity.

Fermentation of cRG-I stimulated the production of several health-promoting metabolites, primarily from amino acid catabolism, while reducing levels of certain metabolites, including two potentially harmful linoleic acid derivatives. Notably, the increased production of IPA, even at a low dose (0.3 g/d) of cRG-I, indicates a highly specific activation of the tryptophan catabolic pathway. This effect was stronger and remarkably consistent across all 24 donors at the higher dose of 1.5 g/d cRG-I. The specific increase in IPA production potentially provides systemic health benefits to the host. The anti-inflammatory and antioxidant effects of indole derivatives like IPA have been described in some detail [8,46]. More recently, IPA and its precursor ILA were shown to protect mice from intestinal inflammation in different colitis models [47]. Moreover, IPA and ILA were shown to have a feedback effect on the gut microbiota, promoting the growth of beneficial microbes such as *Bifidobacterium* and *Akkermansia* species [47]. In addition, IPA has been shown in preclinical studies to facilitate nerve generation and functional recovery in a mouse model [48] and notably to mediate gut–brain interactions, reducing neuroinflammation and improving cognitive function in healthy elderly subjects [49]. Further, in humans, lower circulating IPA levels have been associated with diseases in other organs, including liver fibrosis [50], chronic kidney disease (CKD) [51] and atherosclerotic cardiovascular disease [52], supporting the notion that IPA may exert protective effects on these organs. Other interesting beneficial properties of IPA include its association with lower risk of type 2 diabetes [53], potential antitubercular properties [54], protective effects against allergic airway inflammation [55], and its role in promoting skeletal muscle development and reducing muscle inflammation [56].

Interestingly, IAA, which is produced from tryptophan Via a competing oxidative pathway as opposed to the reductive conversion to ILA and IPA (Figure 4f), was not increased by cRG-I (Figure 2). Although IAA also exhibits anti-inflammatory effects, it has been shown to be less effective than ILA and IPA in ameliorating intestinal inflammation in mice [47]. Further, unlike IPA, IAA can undergo decarboxylation, generating pro-oxidative radicals and lipid peroxidation, thus potentially causing undesirable health effects [8,57,58]. Thus, fibers such as cRG-I that specifically increase conversion of tryptophan to IPA and not IAA, may be considered as attractive candidates to develop functional foods or supplements.

Recently, pectin supplementation was shown to increase ILA, IPA as well as IAA by promoting cross-feeding between *Bacteroides thetaiotaomicron* and *Escherichia coli*, leading to catabolic repression of indole production by *E. coli* and diversion of tryptophan resources to Stickland conversion into ILA, IPA and IAA [19] (Figure 4f). Interestingly, cRG-I fermentation also enhanced the abundance of OTUs related to *B. thetaiotaomicron* but not *E. coli*, similar to pectin’s effects on these taxa in mouse fecal samples (Figure S4) [19]. Therefore, catabolic repression of indole biosynthesis Via bacterial cross-feeding could be a contributing factor to increased IPA production upon cRG-I supplementation.

We hypothesize that cRG-I consistently and specifically promoted the production of IPA, rather than IAA via a reductive pathway leading to the conversion of the common intermediate IPyA into ILA, the precursor of IPA (Figure 4f). This process involved *Bifidobacterium longum*, as evidenced by the strong positive correlation between the related OTU and IPA levels across all study arms and donors (Figure 4e), pointing to *B. longum* as a key player in IPA production. While *B. longum* itself is not known to produce IPA directly, *B. longum* contains an aromatic lactate dehydrogenase (ALDH) that can convert tryptophan to ILA [59,60], which potentially cross-feeds IPA-producing species, thereby enhancing IPA production (Figure 4f). Indeed, ILA and a probiotic *Lactobacillus reuteri* strain that produces ILA have recently been shown to increase IPA production in vitro through microbial cross-feeding [47]. Additional evidence for the role of *B. longum* in IPA production includes in vivo preclinical studies demonstrating that administration of a probiotic *B. longum* strain increased serum IPA levels in mice [61]. In line with this result, the administration of a probiotic mix of *B. longum* and *Bifidobacterium bifidum* in elderly subjects established that the IPA produced by the gut microbiota promoted neuronal function [49]. Further, *B. longum* is present in gut microbiota across the whole lifespan and across a very wide age range of geographies [62,63,64]. This suggests that the promotion of *B. longum* by cRG-I, and subsequent stimulation of IPA is unlikely to be restricted by age-specific or geographic factors. Interestingly, the stimulation of other more abundant bifidobacteria, such as *B. adolescentis*, may not significantly stimulate IPA production, as shown by a much weaker correlation between *B. adolescentis* (OTU3) and IPA levels. This is in line with previous findings that *B. adolescentis* monocultures induce only negligible ILA production compared to *B. longum* [59].

Previously, α-diversity in terms of species richness based on Chao1 diversity index was shown to be unaffected by the IN, cRG-I and XA fibers used in this study [15]. However, by applying the recently introduced CMS [24], that addresses the biases of traditional α-diversity indices, the combined score revealed that cRG-I positively influenced microbial diversity, while XA had a negative effect at 1.5 g/L (Figure S5). The same dose of IN exhibited a neutral impact based on the combined CMS, in contrast to a rather negative effect observed with a higher dose of 5 g/d in a previous study [24] (at such high dose, IN was shown to inhibit acid intolerant gut microbes). The enhanced microbial diversity observed for cRG-I, as indicated by the positive CMS score, may be an important factor contributing to increased production of IPA, corresponding with earlier observations that gut microbiome α-diversity is positively associated with serum IPA levels in a cohort of elderly subjects [65]. Since *B. longum* itself cannot produce IPA, a high microbial diversity potentially ensures the presence of IPA-producing community members that can engage in cross-feeding with *B. longum*, a mechanism that could be further elucidated in vitro. It will also be important to confirm that dietary intake of cRG-I by healthy human subjects leads to increased circulating levels of IPA.

## 5. Conclusions

cRG-I was previously shown to strengthen the immune defenses against a common cold virus in healthy humans [66,67]. This was mediated by a direct effect on the immune system and the beneficial modulation of the gut microbiota. In the current study, we show that cRG-I specifically and consistently steered tryptophan metabolism towards reductive conversion into IPA by increasing the abundance of ILA-producing *B. longum* as well as positively affecting microbial diversity in vitro. These findings are congruent with published preclinical and clinical studies and demonstrate that low doses of cRG-I supplementation sustain the production of enhanced levels of various health-related amino acid catabolites, like IPA, as well as health-beneficial SCFAs. The SIFR^®^ technology used in this study generated biorelevant insights into the mode of action of dietary interventions and their impact on the microbiome function, which supports the design of dedicated future human intervention studies.

## Data Availability

The datasets generated during and/or analyzed during the current study are available from the corresponding author upon reasonable request.

## Supplementary Materials

The following supporting information can be downloaded at: https://www.mdpi.com/article/10.3390/metabo14120722/s1, Figure S1: Correlations established between microbial composition and metabolite production; Figure S2: Representative MS/MS spectrum of the tryptophan derivative indole-3-propionic acid (IPA); Figure S3: *B. adolescentis* abundances and IPA levels displayed a relatively low positive correlation; Figure S4: The effects of cRG-I, XA and IN fermentation on *Bacteroides thetaiotaomicron* (OTU7) and *Escherichia coli* (OTU2); Figure S5: cRG-I exerted a more positive impact on microbial diversity compared to XA and IN.

## References

[1] Krautkramer K.A., Fan J., Bäckhed F. (2021). Gut Microbial Metabolites as Multi-Kingdom Intermediates. Nat. Rev. Microbiol..

[2] Li T.-T., Chen X., Huo D., Arifuzzaman M., Qiao S., Jin W.-B., Shi H., Li X.V., Iliev I.D., Artis D. (2024). Microbiota Metabolism of Intestinal Amino Acids Impacts Host Nutrient Homeostasis and Physiology. Cell Host Microbe.

[3] Saxami G., Kerezoudi E., Eliopoulos C., Arapoglou D., Kyriacou A. (2023). The Gut–Organ Axis within the Human Body: Gut Dysbiosis and the Role of Prebiotics. Life.

[4] Roager H.M., Licht T.R. (2018). Microbial Tryptophan Catabolites in Health and Disease. Nat. Commun..

[5] Li X., Zhang B., Hu Y., Zhao Y. (2021). New Insights Into Gut-Bacteria-Derived Indole and Its Derivatives in Intestinal and Liver Diseases. Front. Pharmacol..

[6] Romagnoli P.A., Shenk G.K., Pham Q.-M., Maher L., Khanna K.M. (2016). Commensal Metabolite Indol-3-Propionic Acid Promotes Gut Barrier Function by Regulating IL-22 Production during Intestinal Inflammatory Conditions. J. Immunol..

[7] Lamas B., Natividad J.M., Sokol H. (2018). Aryl Hydrocarbon Receptor and Intestinal Immunity. Mucosal Immunol..

[8] Jiang H., Chen C., Gao J. (2022). Extensive Summary of the Important Roles of Indole Propionic Acid, a Gut Microbial Metabolite in Host Health and Disease. Nutrients.

[9] Konopelski P., Mogilnicka I. (2022). Biological Effects of Indole-3-Propionic Acid, a Gut Microbiota-Derived Metabolite, and Its Precursor Tryptophan in Mammals’ Health and Disease. Int. J. Mol. Sci..

[10] Zhang P. (2022). Influence of Foods and Nutrition on the Gut Microbiome and Implications for Intestinal Health. Int. J. Mol. Sci..

[11] Gibson G.R., Hutkins R., Sanders M.E., Prescott S.L., Reimer R.A., Salminen S.J., Scott K., Stanton C., Swanson K.S., Cani P.D. (2017). Expert Consensus Document: The International Scientific Association for Probiotics and Prebiotics (ISAPP) Consensus Statement on the Definition and Scope of Prebiotics. Nat. Rev. Gastroenterol. Hepatol..

[12] Carlson J.L., Erickson J.M., Lloyd B.B., Slavin J.L. (2018). Health Effects and Sources of Prebiotic Dietary Fiber. Curr. Dev. Nutr..

[13] Cantu-Jungles T.M., Bulut N., Chambry E., Ruthes A., Iacomini M., Keshavarzian A., Johnson T.A., Hamaker B.R. (2021). Dietary Fiber Hierarchical Specificity: The Missing Link for Predictable and Strong Shifts in Gut Bacterial Communities. mBio.

[14] Cantu-Jungles T.M., Hamaker B.R. (2020). New View on Dietary Fiber Selection for Predictable Shifts in Gut Microbiota. mBio.

[15] Van den Abbeele P., Deyaert S., Albers R., Baudot A., Mercenier A. (2023). Carrot RG-I Reduces Interindividual Differences between 24 Adults through Consistent Effects on Gut Microbiota Composition and Function ex vivo. Nutrients.

[16] Blaak E.E., Canfora E.E., Theis S., Frost G., Groen A.K., Mithieux G., Nauta A., Scott K., Stahl B., Van Harsselaar J. (2020). Short Chain Fatty Acids in Human Gut and Metabolic Health. Benef. Microbes.

[17] Tan J., McKenzie C., Potamitis M., Thorburn A.N., Mackay C.R., Macia L. (2014). The Role of Short-Chain Fatty Acids in Health and Disease. Adv. Immunol..

[18] Huang Z., Wells J.M., Fogliano V., Capuano E. (2024). Microbial Tryptophan Catabolism as an Actionable Target via Diet-Microbiome Interactions. Crit. Rev. Food Sci. Nutr..

[19] Sinha A.K., Laursen M.F., Brinck J.E., Rybtke M.L., Hjørne A.P., Procházková N., Pedersen M., Roager H.M., Licht T.R. (2024). Dietary Fibre Directs Microbial Tryptophan Metabolism via Metabolic Interactions in the Gut Microbiota. Nat. Microbiol..

[20] Van den Abbeele P., Verstrepen L., Ghyselinck J., Albers R., Marzorati M., Mercenier A. (2020). A Novel Non-Digestible, Carrot-Derived Polysaccharide (CRG-I) Selectively Modulates the Human Gut Microbiota While Promoting Gut Barrier Integrity: An Integrated in vitro Approach. Nutrients.

[21] Van den Abbeele P., Deyaert S., Thabuis C., Perreau C., Bajic D., Wintergerst E., Joossens M., Firrman J., Walsh D., Baudot A. (2023). Bridging Preclinical and Clinical Gut Microbiota Research Using the ex ivo SIFR^®^ Technology. Front. Microbiol..

[22] Van den Abbeele P., Kunkler C.N., Poppe J., Rose A., van Hengel I.A.J., Baudot A., Warner C.D. (2024). Serum-Derived Bovine Immunoglobulin Promotes Barrier Integrity and Lowers Inflammation for 24 Human Adults ex vivo. Nutrients.

[23] Van den Abbeele P., Poppe J., Deyaert S., Laurie I., Otto Gravert T.K., Abrahamsson A., Baudot A., Karnik K., Risso D. (2023). Low-No-Calorie Sweeteners Exert Marked Compound-Specific Impact on the Human Gut Microbiota ex vivo. Int. J. Food Sci. Nutr..

[24] Tintoré M., Cuñé J., Vu L.D., Poppe J., Van den Abbeele P., Baudot A., de Lecea C. (2024). A Long-Chain Dextran Produced by Weissella Cibaria Boosts the Diversity of Health-Related Gut Microbes ex vivo. Biology.

[25] Rohart F., Gautier B., Singh A., Cao K.-A.L. (2017). MixOmics: An R Package for ‘omics Feature Selection and Mul-tiple Data Integration. PLOS Comput. Biol..

[26] Chappell C.L., Hoffman K.L., Lorenzi P.L., Tan L., Petrosino J.F., Gibbs R.A., Muzny D.M., Doddapaneni H., Ross M.C., Menon V.K. (2023). Tryptophan Metabolites and Their Predicted Microbial Sources in Fecal Samples of Healthy Individuals. bioRxiv.

[27] Kitada Y., Muramatsu K., Toju H., Kibe R., Benno Y., Kurihara S., Matsumoto M. (2018). Bioactive Polyamine Production by a Novel Hybrid System Comprising Multiple Indigenous Gut Bacterial Strategies. Sci. Adv..

[28] Dolecka J., Urbanik-Sypniewska T., Skrzydło-Radomańska B., Parada-Turska J. (2011). Effect of Kynurenic Acid on the Viability of Probiotics in vitro. Pharmacol. Rep..

[29] Noda H., Akasaka N., Ohsugi M. (1994). Biotin Production by Bifidobacteria. J. Nutr. Sci. Vitaminol..

[30] Hirano R., Shirasawa H., Kurihara S. (2021). Health-Promoting Effects of Dietary Polyamines. Med. Sci..

[31] Eisenberg T., Abdellatif M., Schroeder S., Primessnig U., Stekovic S., Pendl T., Harger A., Schipke J., Zimmermann A., Schmidt A. (2016). Cardioprotection and Lifespan Extension by the Natural Polyamine Spermidine. Nat. Med..

[32] Nilsson L.M., Green L.C., Muralidharan S.V., Demir D., Welin M., Bhadury J., Logan D.T., Walse B., Nilsson J.A. (2016). Cancer Differentiating Agent Hexamethylene Bisacetamide Inhibits BET Bromodomain Proteins. Cancer Res..

[33] Gamage A.M., Lee K.-O., Gan Y.-H. (2017). Anti-Cancer Drug HMBA Acts as an Adjuvant during Intracellular Bacterial Infections by Inducing Type I IFN through STING. J. Immunol..

[34] Park S., Oh S., Kim N., Kim E. (2023). HMBA Ameliorates Obesity by MYH9- and ACTG1-dependent Regulation of Hypothalamic Neuropeptides. EMBO Mol. Med..

[35] Saleem T.H., Abo El-Maali N., Hassan M.H., Mohamed N.A., Mostafa N.A.M., Abdel-Kahaar E., Tammam A.S. (2018). Comparative Protective Effects of N-Acetylcysteine, N-Acetyl Methionine, and N-Acetyl Glucosamine against Paracetamol and Phenacetin Therapeutic Doses–Induced Hepatotoxicity in Rats. Int. J. Hepatol..

[36] Hwang I.-Y., Jeong C.-S. (2012). Inhibitory Effects of 4-Guanidinobutyric Acid against Gastric Lesions. Biomol. Ther..

[37] Li H., Xiao H., Yuan L., Yan B., Pan Y., Tian P., Zhang W. (2023). Protective Effect of L-Pipecolic Acid on Constipation in C57BL/6 Mice Based on Gut Microbiome and Serum Metabolomic. BMC Microbiol..

[38] Hildreth K., Kodani S.D., Hammock B.D., Zhao L. (2020). Cytochrome P450-Derived Linoleic Acid Metabolites EpOMEs and DiHOMEs: A Review of Recent Studies. J. Nutr. Biochem..

[39] Levan S.R., Stamnes K.A., Lin D.L., Panzer A.R., Fukui E., McCauley K., Fujimura K.E., McKean M., Ownby D.R., Zoratti E.M. (2019). Elevated Faecal 12,13-DiHOME Concentration in Neonates at High Risk for Asthma Is Produced by Gut Bacteria and Impedes Immune Tolerance. Nat. Microbiol..

[40] Duan J., Dong W., Wang G., Xiu W., Pu G., Xu J., Ye C., Zhang X., Zhu Y., Wang C. (2023). Senescence-Associated 13-HODE Production Promotes Age-Related Liver Steatosis by Directly Inhibiting Catalase Activity. Nat. Commun..

[41] Mabalirajan U., Rehman R., Ahmad T., Kumar S., Singh S., Leishangthem G.D., Aich J., Kumar M., Khanna K., Singh V.P. (2013). Linoleic Acid Metabolite Drives Severe Asthma by Causing Airway Epithelial Injury. Sci. Rep..

[42] Huang J., Zhao B., Weinstein S.J., Albanes D., Mondul A.M. (2022). Metabolomic Profile of Prostate Cancer-Specific Survival among 1812 Finnish Men. BMC Med..

[43] Vaezi M.A., Safizadeh B., Eghtedari A.R., Ghorbanhosseini S.S., Rastegar M., Salimi V., Tavakoli-Yaraki M. (2021). 15-Lipoxygenase and Its Metabolites in the Pathogenesis of Breast Cancer: A Double-Edged Sword. Lipids Health Dis..

[44] Chang J., Jiang L., Wang Y., Yao B., Yang S., Zhang B., Zhang M.-Z. (2015). 12/15 Lipoxygenase Regulation of Colorectal Tumorigenesis Is Determined by the Relative Tumor Levels of Its Metabolite 12-HETE and 13-HODE in Animal Models. Oncotarget.

[45] Jian C., Sorensen N., Lutter R., Albers R., De Vos W., Salonen A., Mercenier A. (2024). The Impact of Daily Sup-plementation with Rhamnogalacturonan-I on the Gut Microbiota in Healthy Adults: A Randomized Con-trolled Trial. Biomed. Pharmacother..

[46] Negatu D., Gengenbacher M., Dartois V., Dick T. (2020). Indole Propionic Acid, an Unusual Antibiotic Produced by the Gut Microbiota, with Anti-Inflammatory and Antioxidant Properties. Front. Microbiol..

[47] Wang G., Fan Y., Zhang G., Cai S., Ma Y., Yang L., Wang Y., Yu H., Qiao S., Zeng X. (2024). Microbiota-Derived Indoles Alleviate Intestinal Inflammation and Modulate Microbiome by Microbial Cross-Feeding. Microbiome.

[48] Serger E., Luengo-Gutierrez L., Chadwick J.S., Kong G., Zhou L., Crawford G., Danzi M.C., Myridakis A., Brandis A., Bello A.T. (2022). The Gut Metabolite Indole-3 Propionate Promotes Nerve Regeneration and Repair. Nature.

[49] Kim C.-S., Jung S., Hwang G.-S., Shin D.-M. (2023). Gut Microbiota Indole-3-Propionic Acid Mediates Neuroprotective Effect of Probiotic Consumption in Healthy Elderly: A Randomized, Double-Blind, Placebo-Controlled, Multicenter Trial and in vitro Study. Clin. Nutr..

[50] Sehgal R., Ilha M., Vaittinen M., Kaminska D., Männistö V., Kärjä V., Tuomainen M., Hanhineva K., Romeo S., Pajukanta P. (2021). Indole-3-Propionic Acid, a Gut-Derived Tryptophan Metabolite, Associates with Hepatic Fibrosis. Nutrients.

[51] Sun C.-Y., Lin C.-J., Pan H.-C., Lee C.-C., Lu S.-C., Hsieh Y.-T., Huang S.-Y., Huang H.-Y. (2019). Clinical Association between the Metabolite of Healthy Gut Microbiota, 3-Indolepropionic Acid and Chronic Kidney Disease. Clin. Nutr..

[52] Xue H., Chen X., Yu C., Deng Y., Zhang Y., Chen S., Chen X., Chen K., Yang Y., Ling W. (2022). Gut Microbially Produced Indole-3-Propionic Acid Inhibits Atherosclerosis by Promoting Reverse Cholesterol Transport and Its Deficiency Is Causally Related to Atherosclerotic Cardiovascular Disease. Circ. Res..

[53] Tuomainen M., Lindström J., Lehtonen M., Auriola S., Pihlajamäki J., Peltonen M., Tuomilehto J., Uusitupa M., De Mello V.D., Hanhineva K. (2018). Associations of Serum Indolepropionic Acid, a Gut Microbiota Metabolite, with Type 2 Diabetes and Low-Grade Inflammation in High-Risk Individuals. Nutr. Diabetes.

[54] Negatu D.A., Liu J.J.J., Zimmerman M., Kaya F., Dartois V., Aldrich C.C., Gengenbacher M., Dick T. (2018). Whole-Cell Screen of Fragment Library Identifies Gut Microbiota Metabolite Indole Propionic Acid as Antitubercular. Antimicrob. Agents Chemother..

[55] Perdijk O., Butler A., Macowan M., Chatzis R., Bulanda E., Grant R.D., Harris N.L., Wypych T.P., Marsland B.J. (2024). Antibiotic-Driven Dysbiosis in Early Life Disrupts Indole-3-Propionic Acid Production and Exacerbates Allergic Airway Inflammation in Adulthood. Immunity.

[56] Du L., Qi R., Wang J., Liu Z., Wu Z. (2021). Indole-3-Propionic Acid, a Functional Metabolite of *Clostridium sporogenes*, Promotes Muscle Tissue Development and Reduces Muscle Cell Inflammation. Int. J. Mol. Sci..

[57] Candeias L.P., Folkes L.K., Porssa M., Parrick J., Wardman P. (1995). Enhancement of Lipid Peroxidation by Indole-3-Acetic Acid and Derivatives: Substituent Effects. Free Radic. Res..

[58] Cuevas-Gómez I., De Andrés J., Cardenas N., Espinosa-Martos I., Jiménez E. (2022). Safety Assessment and Characterisation of *Ligilactobacillus salivarius* PS21603 as Potential Feed Additive for Swine. Benef. Microbes.

[59] Sakurai T., Odamaki T., Xiao J. (2019). Production of Indole-3-Lactic Acid by *Bifidobacterium* Strains Isolated From Human Infants. Microorganisms.

[60] Laursen M.F., Sakanaka M., von Burg N., Mörbe U., Andersen D., Moll J.M., Pekmez C.T., Rivollier A., Michaelsen K.F., Mølgaard C. (2021). *Bifidobacterium* Species Associated with Breastfeeding Produce Aromatic Lactic Acids in the Infant Gut. Nat. Microbiol..

[61] Rätsep M., Kilk K., Zilmer M., Kuusik S., Kuus L., Vallas M., Gerulis O., Štšepetova J., Orav A., Songisepp E. (2024). Investigation of Effects of Novel *Bifidobacterium longum* ssp. *longum* on Gastrointestinal Microbiota and Blood Serum Parameters in a Conventional Mouse Model. Microorganisms.

[62] Lu J., Zhang L., Zhang H., Chen Y., Zhao J., Chen W., Lu W., Li M. (2023). Population-Level Variation in Gut Bifidobacterial Composition and Association with Geography, Age, Ethnicity, and Staple Food. npj Biofilms Microbiomes.

[63] Oki K., Akiyama T., Matsuda K., Gawad A., Makino H., Ishikawa E., Oishi K., Kushiro A., Fujimoto J. (2018). Long-term colonization exceeding six years from early infancy of *Bifidobacterium longum* subsp. longum in human gut. BMC Microbiol..

[64] Nagpal R., Kurakawa T., Tsuji H., Takahashi T., Kawashima K., Nagata S., Nomoto K., Yamashiro Y. (2017). Evolution of Gut *Bifidobacterium* Population in Healthy Japanese Infants over the First Three Years of Life: A Quantitative Assessment. Sci. Rep..

[65] Menni C., Hernandez M.M., Vital M., Mohney R.P., Spector T.D., Valdes A.M. (2019). Circulating Levels of the Anti-oxidant Indoleproprionic Acid Are Associated with Higher Gut Microbiome Diversity. Gut Microbes.

[66] Lutter R., Teitsma-Jansen A., Floris E., Lone-Latif S., Ravi A., Sabogal Pineros Y.S., Dekker T., Smids B., Khurshid R., Aparicio-Vergara M. (2021). The Dietary Intake of Carrot-Derived Rhamnogalacturonan-I Accelerates and Augments the Innate Immune and Anti-Viral Interferon Response to Rhinovirus Infection and Reduces Duration and Severity of Symptoms in Humans in a Randomized Trial. Nutrients.

[67] McKay S., Teitsma-Jansen A., Floris E., Dekker T., Smids B., Khurshid R., Calame W., Kardinaal A., Lutter R., Albers R. (2022). Effects of Dietary Supplementation with Carrot-Derived Rhamnogalacturonan-I (CRG-I) on Accelerated Protective Immune Responses and Quality of Life in Healthy Volunteers Challenged with Rhinovirus in a Randomized Trial. Nutrients.

