# Supplementary material for: Carrot-Derived Rhamnogalacturonan-I Consistently Increases the Microbial Production of Health-Promoting Indole-3-Propionic Acid Ex Vivo
Source: Metabolites. 2024 Dec 21;14(12):722. doi: 10.3390/metabo14120722 (PMC11678764; doi:10.3390/metabo14120722)
Supplement: Supplementary file 1 [file metabolites-14-00722-s001.zip › metabolites-3319526-supplementary.pdf]

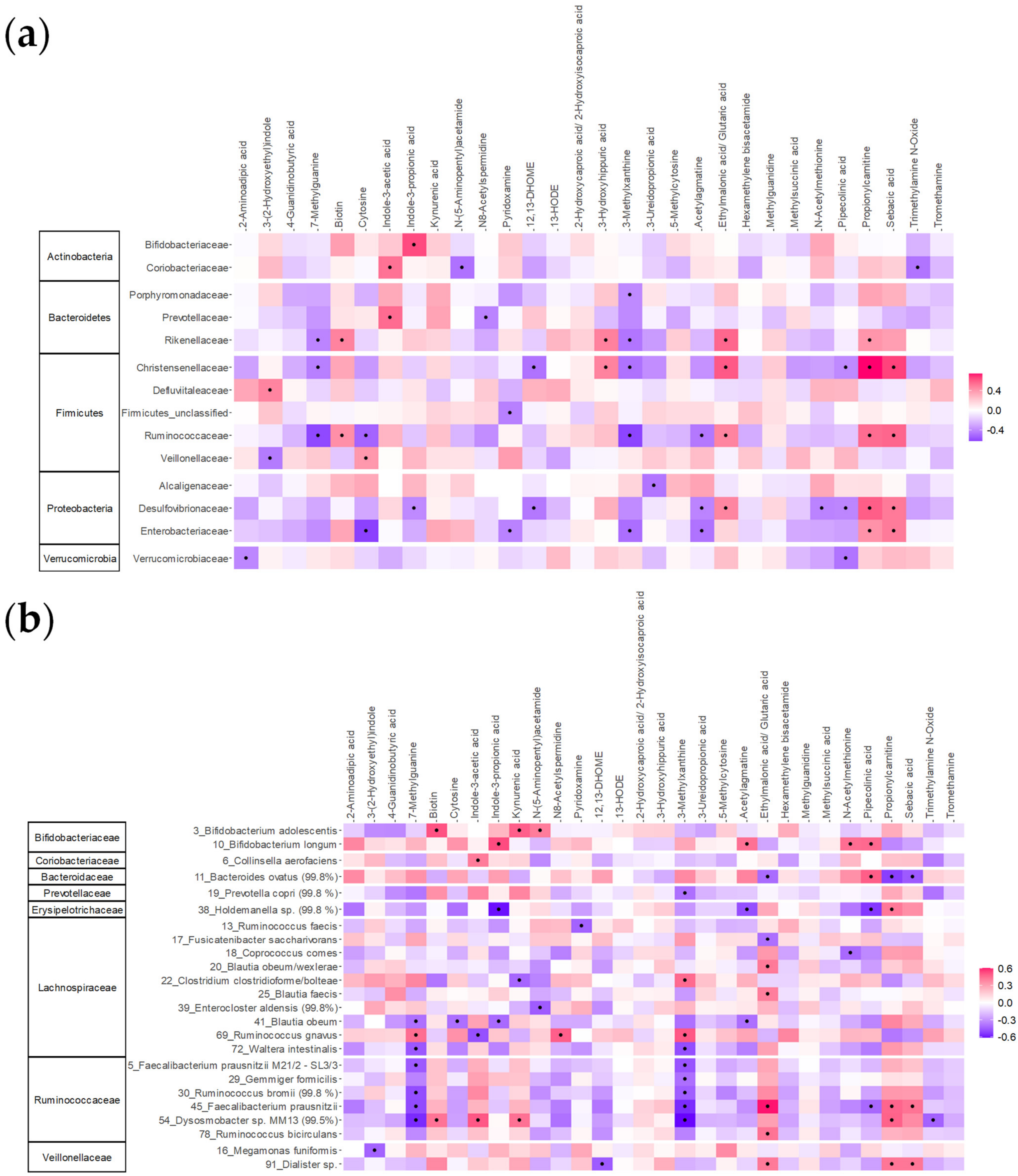

Figure S1. Correlations established between microbial composition and metabolite production. (a) rCCA analysis was performed based on significantly affected metabolites and (**a**) significantly affected bacterial families and (**b**) OTUs. The black dots highlight correlations above the threshold of 0.4.

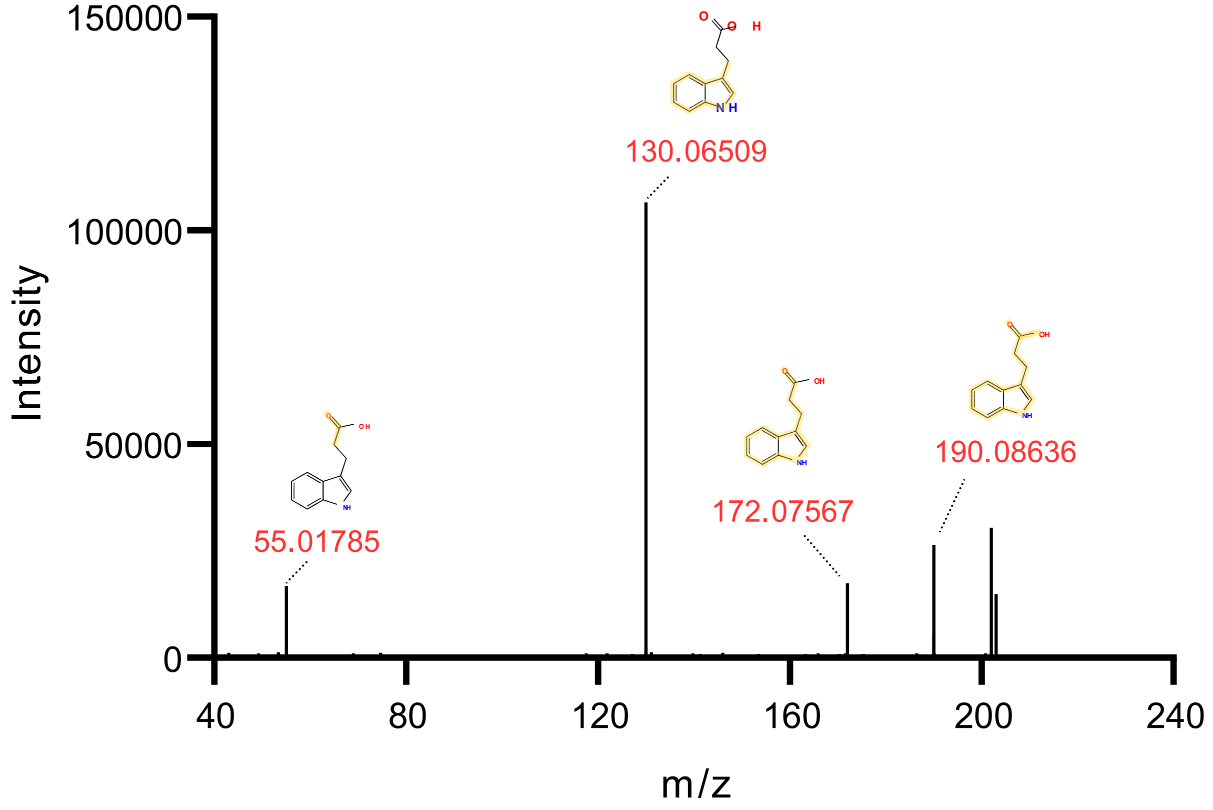

Figure S2. Representative MS/MS spectrum of the tryptophan derivative indole-3-propionic acid (IPA). IPA was identified at level 1, (based on retention times (compared against in-house authentic standards), accurate mass (with an accepted deviation of 3 ppm), and MS/MS spectra). m/z of characteristic fragment ions that matched with the library are shown is indicated in red, together with the corresponding fragment highlighted in yellow.

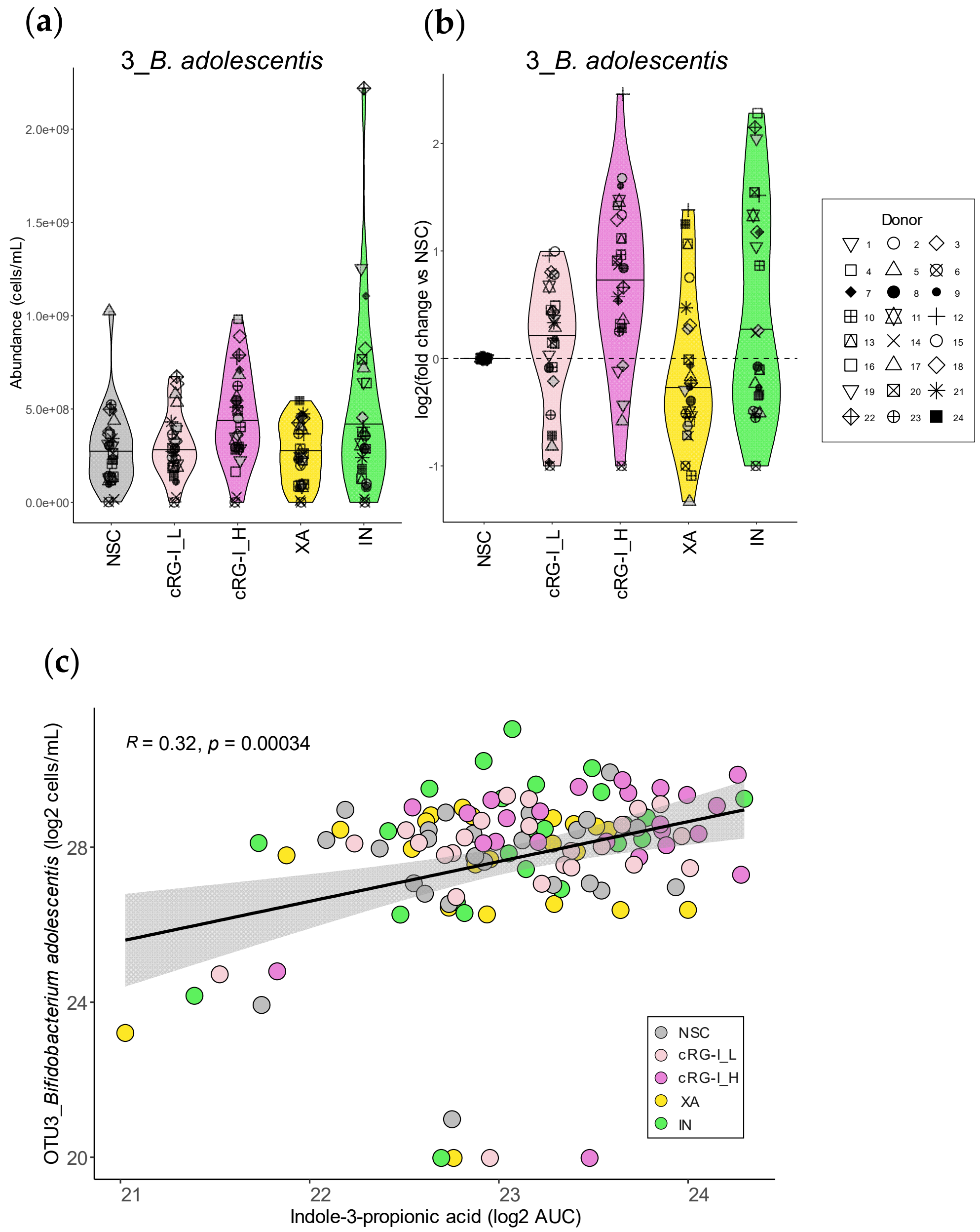

Figure S3. ***B. adolescentis*** **abundances and IPA levels displayed a relatively low positive correlation.** (**a**) Absolute abundances of *Bifidobacterium adolescentis* (OTU3) (in cells/mL) after 48-hour SIFR® colonic fermentation of cRG-I, XA and IN by the gut microbiota of 24 healthy adults compared to the NSC. (**b**) Log_2-transformed fold change of *B. adolescentis* (OTU3) abundance upon fermentation of c-RG-I, XA, IN versus NSC at 48 h. (**c**) Pearson correlation analysis based on log_2-transformed *B. adolescentis* (OTU3) abundances and log_2-transformed IPA levels across all study arms at 48 h. Pearson correlation coefficient (R) and *p*-value indicating the significance of the correlation are presented on the plot.

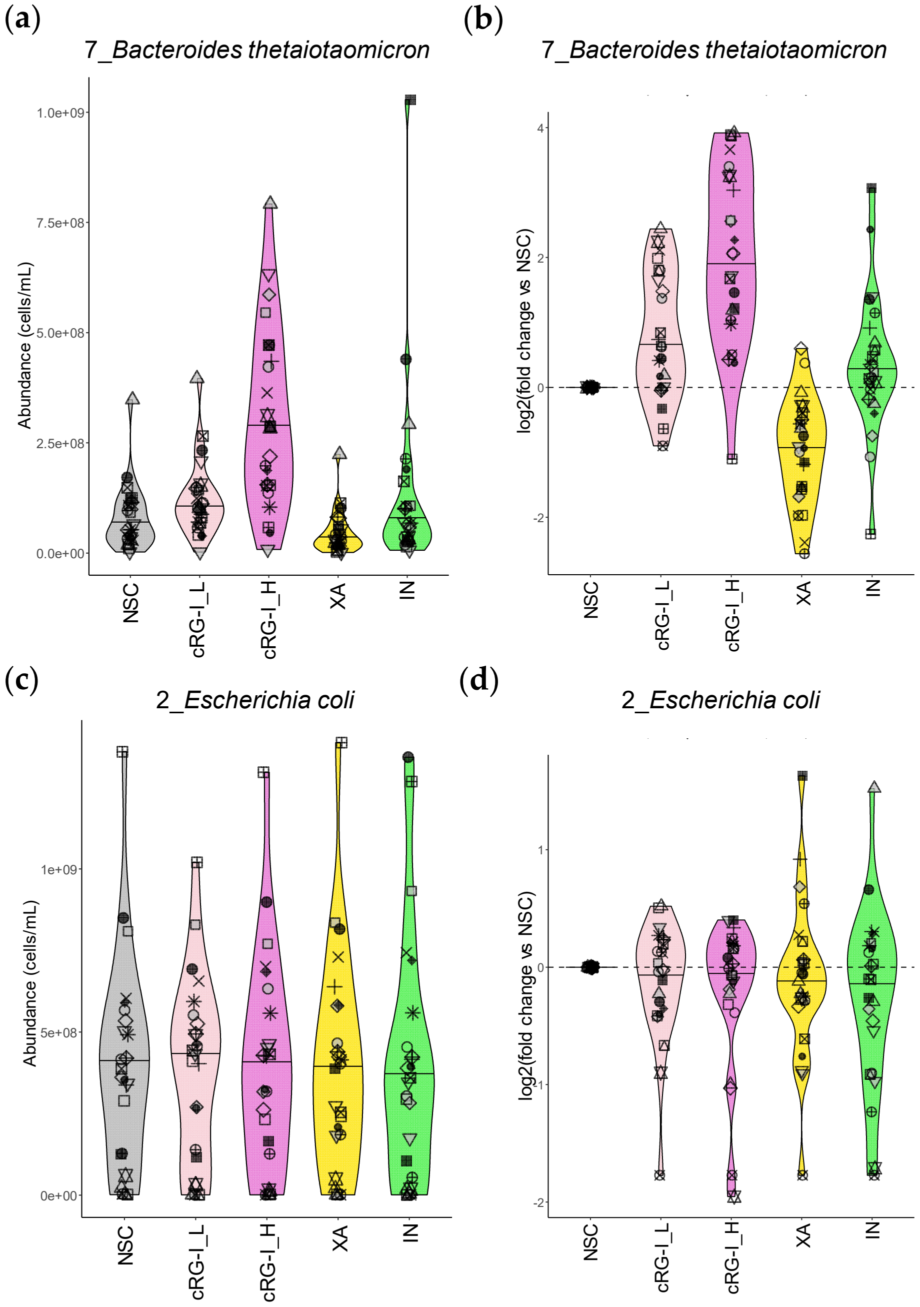

Figure S4. The effects of cRG-I, XA and IN fermentation on *Bacteroides thetaiotaomicron* (OTU7) and *Escherichia coli* (OTU2). (**a**), (**c**) Absolute abundances of the *B. thetaiotaomicron* (OTU7) and *E. coli* (OTU2) (in cells/mL), respectively, after 48-hour SIFR® colonic fermentation of cRG-I, XA and IN by the gut microbiota of 24 healthy adults compared to the NSC. (**b**), (**d**) Log$_2$-transformed fold change of the abundance of the respective OTUs upon fermentation of c-RG-I, XA, IN versus NSC at 48 h.

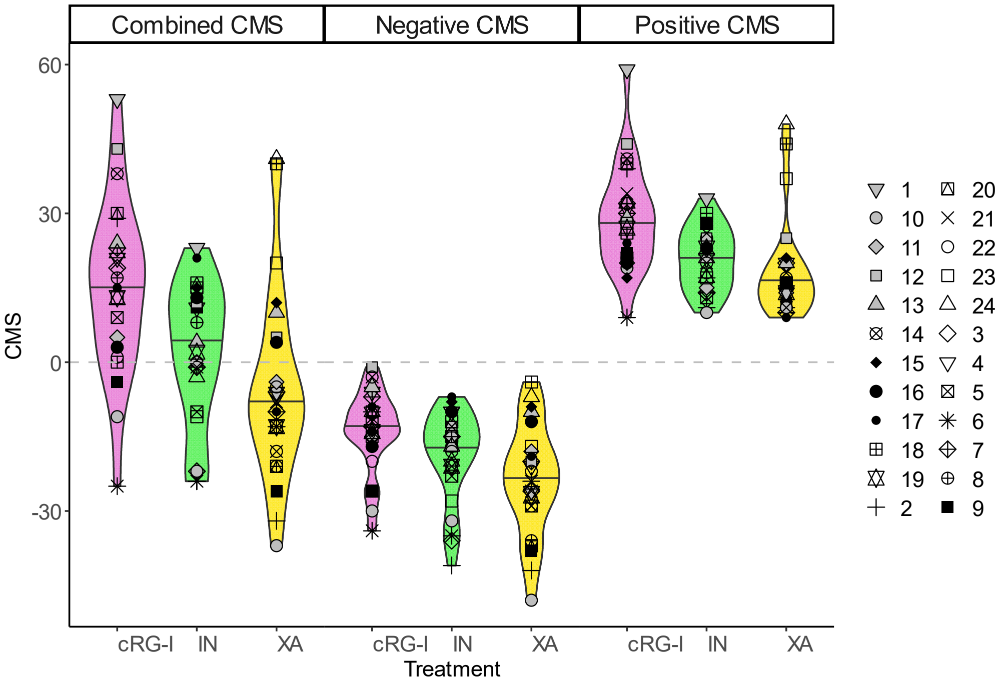

Figure S5. cRG-I exerted a more positive impact on microbial diversity compared to XA and IN. The effects of 1.5 g/d cRG-I, IN and XA on microbial diversity were assessed based on the novel community modulation scores (CMS). CMS was presented as a positive (increased OTUs), negative (decreased OTUs) and combined score (net increased OTUs).
